# Short-term budget affordability of hepatitis C treatments for state Medicaid programs

**DOI:** 10.1186/s12913-019-3956-x

**Published:** 2019-02-28

**Authors:** Jacquelyn W. Chou, Alison R. Silverstein, Dana P. Goldman

**Affiliations:** Precision Health Economics, 11100 Santa Monica Boulevard, Suite 500, Los Angeles, CA 90025 USA

**Keywords:** Hepatitis C, HCV, Access, Medicaid, Value, Model, QALY, Long-term benefits

## Abstract

**Background:**

With some Medicaid state programs still restricting patient access to hepatitis C (HCV) treatment, it is important to demonstrate how states could expand treatment access to a broader Medicaid population and balance short-term budget concerns.

**Methods:**

We used the HCV Transmission and Progression (TaP) Markov model to quantify the impact of removing restrictions to HCV treatment access on the infected populations, expenditures, and net social value for the North Carolina (NC), Oregon (OR), and Wisconsin (WI) Medicaid programs. Four HCV treatment access scenarios were modeled: 1) *Baseline*: Patients were treated according to Medicaid disease severity and sobriety requirements in 2015; 2) *Remove Sobriety Restrictions*: Disease severity restrictions were maintained, but people who inject drugs (PWID) were given access to treatment; 3) *Treat Early*: All patients, except for PWIDs, regardless of disease severity, were eligible for treatment and the diagnosis rate increased from 50 to 66%; and 4) *Remove Access Restrictions*: all patients, regardless of disease severity and sobriety, were eligible for treatment. Our key model outputs were: number of infected Medicaid beneficiaries, HCV-related medical and treatment expenditures, total social value, and state Medicaid spending over 10 years.

**Results:**

Across all three states, removing access restrictions resulted in the greatest benefits over 10 years (net social value relative to baseline = $408 M in NC; $408 M in OR; $271 M in WI) and the smallest infected population (5200 in NC; 2000 in OR; 614 in WI). Reduced disease transmission resulted in lower health care expenditures (-$66 M in NC; -$50 M in OR; -$54 M in WI). All of the expanded treatment access policies achieved break-even costs—where total treatment and health care expenditures fell below those of Baseline—in 4 to 8 years. Removing access restrictions yielded the greatest improvement in social value (net of medical expenditures and treatment costs, QALYs valued at $150 K per QALY).

**Conclusions:**

While increasing treatment access in Medicaid will raise short-term costs, it will also provide clear benefits relatively quickly by saving money and improving health within a 10-year window. Patients and taxpayers would benefit by considering these gains and taking a more expansive and long-term view of HCV treatment policies.

**Electronic supplementary material:**

The online version of this article (10.1186/s12913-019-3956-x) contains supplementary material, which is available to authorized users.

## Background

One of the key challenges in healthcare is that much of the spending is government-financed through Medicaid and Medicare, and therefore subject to short-term fiscal constraints, whereas the benefits from treatments and interventions accrue over much longer time horizons [1]. For instance, when the Congressional Budget Office produces budget projections meant to inform government planning decisions, these projections are conducted over a 10-year time horizon [2]. However, 10 years may only capture a fraction of the benefit of a treatment or other healthcare investment. As an example, in hepatitis C (HCV), progression of mild liver damage to severe or end-stage liver disease often takes place over several decades [3].

Short-term thinking is particularly acute when looking at policy decision-making at the state level where time horizons for budget impact assessments are 1 or 2 years. The outcome of evaluating healthcare investments on a time horizon too short to fully capture the return on investment can result in limiting patient access to valuable healthcare innovations. The limitations of short-term budget modeling can be seen in the case of HCV treatment when direct-acting antiviral (DAA) therapies first came to market. Facing a growing affected population and rising costs of care for HCV, many state Medicaid programs have restricted access to effective, but more costly, DAA therapies used to treat HCV infection [4]. While these therapies have been demonstrated to cure HCV infection in 90–100% of treated patients [5], a 2014 survey of state Medicaid fee-for-service (FFS) programs conducted by the National Viral Hepatitis Roundtable and the Center for Health Law and Policy Innovation of Harvard Law School found a number of treatment access restrictions based on disease severity, sobriety status, and prescriber eligibility [6]. In response, the Centers for Medicare and Medicaid Services (CMS) issued a letter to the states warning that these access restrictions may violate statutory requirements for Medicaid [4].

A follow-up survey found that 18 states had eliminated their disease severity restrictions as of mid-2017, a number that has since grown with states such as Colorado and Washington recently lifting restrictions [6]. Most states (40, according to the report), however, maintained sobriety requirements, ranging from screening and counseling to 12-month abstinence. Though treatment costs have decreased substantially from initial launch prices, state Medicaid programs that maintain access restrictions often indicate cost as the primary driver for limiting treatment [7].

State Medicaid policies for HCV treatment can have a big impact on public health, as HCV disproportionately impacts poor and vulnerable populations that are more likely to access healthcare through state Medicaid programs, including people who inject drugs (PWID) [8]. In fact, it has been estimated that the prevalence of HCV among Medicaid enrollees is 7.5 times higher than the prevalence in the commercially insured population [9]. Cases of new HCV infections have been growing rapidly over the past decade, and the CDC estimated that there were 41,200 new HCV infections in 2016 [10]. Rates of HCV transmission are particularly high among PWID; treating injection drug users can reduce the transmitting population and therefore future infections [11].

Recent research has demonstrated the nationally aggregated benefits and cost-savings of expanded access to DAA therapies for the Medicaid population [12, 13]. Further, the social value generated by treating HCV infection with DAA therapies has previously been quantified [14]. While some payers and policymakers recognize the benefits of DAAs and support expanded treatment [4], many state Medicaid programs, including North Carolina (NC) and Wisconsin (WI), continue to maintain sobriety requirements, while states like Oregon (OR) have recently lifted all restrictions.

In this study, we seek to demonstrate that states can expand DAA treatment access to a broader Medicaid population while balancing short-term budget concerns by quantifying the effect of broader treatment access strategies on the infected population, social value, and budget for the NC, OR, and WI state Medicaid programs.

## Methods

### Model framework

**Fig. 1:**
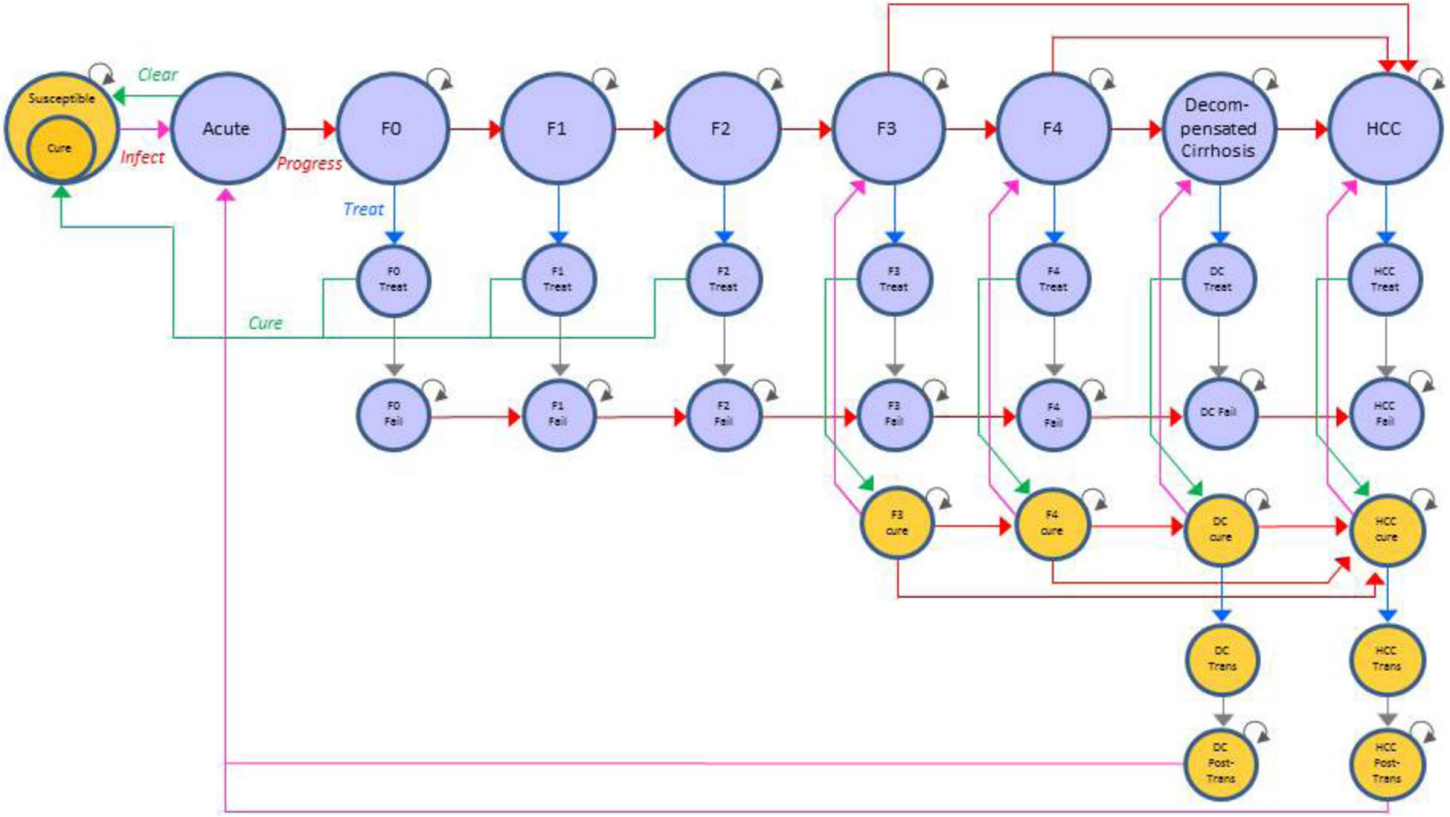
Hepatitis C Transmission and Progression Model Schematic. Notes: Original figure copyrighted and published by Project HOPE/Health Affairs as Karen Van Nuys, Ronald Brookmeyer, Jacquelyn W. Chou, David Dreyfus, Douglas Dieterich, Dana P. Goldman. “Broad hepatitis C treatment scenarios return substantial health gains, but capacity is a concern,” Exhibit A1 [appendix]. Health Affairs (Millwood). 2015; 34(10):1666–1674. The published article is archived and available online at www.healthaffairs.org/. Reused with permission from Project HOPE/Health Affairs. Notes: All states have transitions to “dead.” “Trans” = Transplant. Red Arrow = Progression; Blue Arrow = Treat; Green Arrow = Cure; Pink Arrow = Transmission. Blue shading of a shape indicates infectious/infected population. Yellow shading of a shape indicates uninfected and susceptible population

The model used for this study is adapted from the discrete-time Markov model of the social value of HCV treatment in the United States (US) developed by Van Nuys and colleagues [14]. The HCV Transmission and Progression (TaP) model (Fig. 1) was used to quantify the disease epidemiology and associated economic impact of different treatment access policy scenarios for Medicaid programs in the three states over a 10-year time horizon starting in 2015. The model addresses the experiences of three exposure groups and the three most common genotypes in the US. Each model cycle is one year.

The model captures the transition across disease severity, characterized by seven stages of liver damage. Metavir scores assess liver damage in five of the stages; F0 describes the least damage (no fibrosis), with increasing damage to F4 (compensated cirrhosis). The remaining two stages are the most severe disease states of decompensated cirrhosis and hepatocellular carcinoma.

### Populations modeled

Three prevalent populations in each state were modeled separately: the “At Risk” population of people born between 1945 and 1965 (the CDC recommends HCV testing for all individuals born in this time frame due to the risk of exposure through the US blood supply during their lifetimes); people who inject drugs (PWID); and HIV-positive men who have sex with men (HIV+/MSM) [15]. The three exposure groups were modeled independently with individuals only belonging to one exposure group throughout the simulation with no cross-transmission between the groups. Similar to the Van Nuys model [14], the At Risk exposure group was assumed to have no on-going transmission due to improvements in the screening of blood products and was assumed to be a closed cohort, shrinking over time due to aging and mortality. The PWID and the HIV+/MSM exposure groups were assumed to stay at a constant size throughout the simulation with a constant mortality rate and on-going entry. These three exposure groups comprise the majority of the prevalent and incident HCV populations in the US, with PWIDs being the largest incident population [15].

Genotypes 1, 2, and 3, the most common genotypes in the US [16], were modeled within each exposure group. Modeling genotypes separately was critical as patients infected with different genotypes respond differently to treatment [17], may progress at different rates [18, 19], and have different mortality risks [20]. In the model, patients were permitted to be infected with one genotype at a time, but once cured, a patient could be re-infected with any of the three genotypes. Incidence of HCV at the state level was estimated using data from state public health department reports [21–23], scaling these values by a factor of 12.3 as described in the widely cited Klevens et al. 2014 publication [24]. Incidence for each state was scaled to a rate per the estimated size of the state-specific population in each exposure group.

We aimed to model the HCV-infected population eligible for Medicaid in states that are representative of different Medicaid treatment and eligibility policies, are geographically diverse, and have high rates of HCV relative to the state population. We also searched for states that had state-specific data on the HCV-infected populations available. Considering these criteria, we chose to model the HCV-infected populations in NC, OR, and WI. While not representative of “average” Medicaid programs in the US, these three states enabled us to simulate the relative impact of varying the restrictiveness of access policies more granularly than scaling a national estimate, due to the availability of state-specific estimates.

State-specific prevalence estimates based on HCV ribonucleic acid (RNA) tests were extracted from a recent publication by Rosenberg et al. [25]. We used the HCV RNA results over HCV antibody results as they better account for the subpopulations of patients who would actually be treated [26]. Additional information on the starting populations for each state can be found in the Technical Appendix (Additional file 1: Tables S1-S4).

### Transmission function

In the PWID and HIV+/MSM groups, for each genotype, the rate at which individuals were infected was modeled dynamically as a function of the number in the exposure group who were currently infected with the given genotype. Additional details on the transmission calculations can be found in Van Nuys et al. [14]. The incidence rates and proportionality constants *K* used in this state Medicaid-adapted model are available in the Technical Appendix (Additional file 1: Table S5).

### Treatment assumptions

All treatment scenarios assumed use of the DAA sofosbuvir + velpatasvir. The “Baseline” scenario for each state followed Medicaid criteria for HCV treatment in 2015, and assumed 50% of infected individuals were formally diagnosed [21–23]. In NC, OR, and WI, treatment was restricted based on more advanced disease severity and sobriety requirements. The first alternative treatment scenario, “Remove Sobriety Restrictions,” expanded treatment access to all PWIDs, while maintaining the same disease severity criteria and proportion of diagnosed patients treated as Baseline. Compared to Baseline, the “Remove Sobriety Restrictions” scenario had a larger eligible population for treatment due to the inclusion of PWIDs. The “Treat Early” scenario expanded the Baseline disease severity criteria for treatment to F0+ and simulated a 66% diagnosis rate, increasing the proportion of patients treated. In Treat Early, no PWIDs were treated due to the sobriety restrictions. “Remove Access Restrictions” used the Treat Early severity criteria and diagnosis rates, but also removed the sobriety restriction, allowing PWIDs to be treated. Compared to Baseline, increasing the proportion of diagnosed patients or removing any treatment severity or sobriety requirements for treatment eligibility increases the absolute number of patients eligible for treatment and therefore also the absolute number of patients treated between scenarios in this model. Baseline and all alternative treatment policy scenarios are shown in Table 1.

**Table 1:** Regimens, Duration, and Efficacy for Four Treatment Scenarios Modeled

		Baseline		Remove Sobriety Restrictions		Treat Early		Remove Access Restrictions	
North Carolina		F2+, Treat 6%, no PWIDs		F2+, Treat 6%, Treat PWIDs		F0+, Treat 8%, No PWIDs		F0+, Treat 8%, Treat PWIDs	
Oregon		F3+, Treat 10%, no PWIDs		F3+, Treat 10%, Treat PWIDs		F0+, Treat 13%, No PWIDs		F0+, Treat 13%, Treat PWIDs	
Wisconsin		F3+, Treat 18%, no PWIDs		F3+, Treat 18%, Treat PWIDs		F0+, Treat 24%, No PWIDs		F0+, Treat 24%, Treat PWIDs	
Drugs used		sofosbuvir + velpatasvir for 12 weeks							
SVR by disease stage		F0-F3	F4, DC, HCC	F0-F3	F4, DC, HCC	F0-F3	F4, DC, HCC	F0-F3	F4, DC, HCC
Genotype 1	At Risk	0.98	1.00	Same as Baseline					
	PWID	0.98	1.00	Same as Baseline					
	HIV+/MSM	0.96	0.96	Same as Baseline					
Genotype 2	At Risk	0.99	1.00	Same as Baseline					
	PWID	0.99	1.00	Same as Baseline					
	HIV+/MSM	1.00	1.00	Same as Baseline					
Genotype 3	At Risk	0.97	0.92	Same as Baseline					
	PWID	0.97	0.92	Same as Baseline					
	HIV+/MSM	0.92	0.91	Same as Baseline					

A weighted sustained virological response (SVR) was calculated in order to account for differences in SVR between treatment-naïve and treatment-experienced patients observed in clinical trials of sofosbuvir + velpatasvir. This weighted SVR was based on 80% of the population never receiving prior treatment and is described in the Technical Appendix (Additional file 1: Table S6).

In all scenarios, a constant proportion of the infected population was treated in each model cycle. After the first model cycle, a large proportion of patients move out of the prevalent population to the susceptible population as a result of being cured. In each following cycle, the diagnosis rate and proportion of infected patients treated are the same as those used in the first cycle.

The wholesale acquisition cost (WAC) of a 12-week course of treatment with sofosbuvir + velpatasvir in 2015 was roughly $75,000. A 43% Medicaid discount [27] was applied to this cost such that the treatment costs modeled were $43,000 per course. Because sofosbuvir + velpatasvir remains under patent protection, a cost trajectory was modeled to account for decreasing price due to increasing market competition. In year 3, the price was reduced to $30,000 per 12-week regimen in state treatment scenarios that expand access to PWIDs (Remove Sobriety Restrictions and Remove Access Restrictions), reflecting pricing for state Medicaid programs who provide open access to all patients [28]. To account for entry of competition, the price for years 4–10 was $20,000 to match a competitor’s pricing. We assume that patent expiration would occur in year 15, which would then reduce prices by 79% from the baseline cost, which is assumed to be the marginal cost of producing the drugs [29]. However, our model ends at year 10, and therefore we do not capture the effect of patent expiration on treatment costs.

Regimen drugs, duration, efficacy, and treatment costs are also detailed in Table 1.

### Model parameters

All model parameters were collected from data obtained through a literature search of published research and a review of publicly available reports for each state’s population, Medicaid program, and health statistics. Details on the sources of model parameters can be found in the Technical Appendix (Additional file 1: Tables S7-S9).

### Model outputs

The outcomes of interest for this analysis were: HCV prevalence; HCV-related health care expenditures inclusive of both treatment and medical costs; and social value. Social value was calculated as quality-adjusted life-years (QALYs) net of HCV-related health care expenditures. Each QALY was valued at $150,000 [30], with alternate values tested in sensitivity analyses, which captures both the life-extension and improved health-related quality of life generated by each treatment scenario.

### State spending analysis

The annual contributions from federal and state sources to the total treatment and medical expenditures produced by the model were estimated to assess the extent to which additional state resources would be needed to fund expanded access. We applied the year- and state-specific Federal Medical Assistance Percentage (FMAP) for Medicaid to total health care expenditures to determine the federal contribution for each year [31], and attributed the remainder of the costs to the state’s spending. The proportion of federal contribution changes year-to-year and was specified for the federal fiscal year (FY), whereas the TaP model runs on the calendar year. FMAPs were only available through FY 2018, so we adjusted the FY FMAP to the calendar year (CY) for CYs 2015–2018 and applied the CY 2018 FMAP to the remaining model years. The FY and CY FMAPs by state can be found in the Technical Appendix (Additional file 1: Table S15).

## Results

### State-Medicaid HCV prevalence

**Fig. 2:**
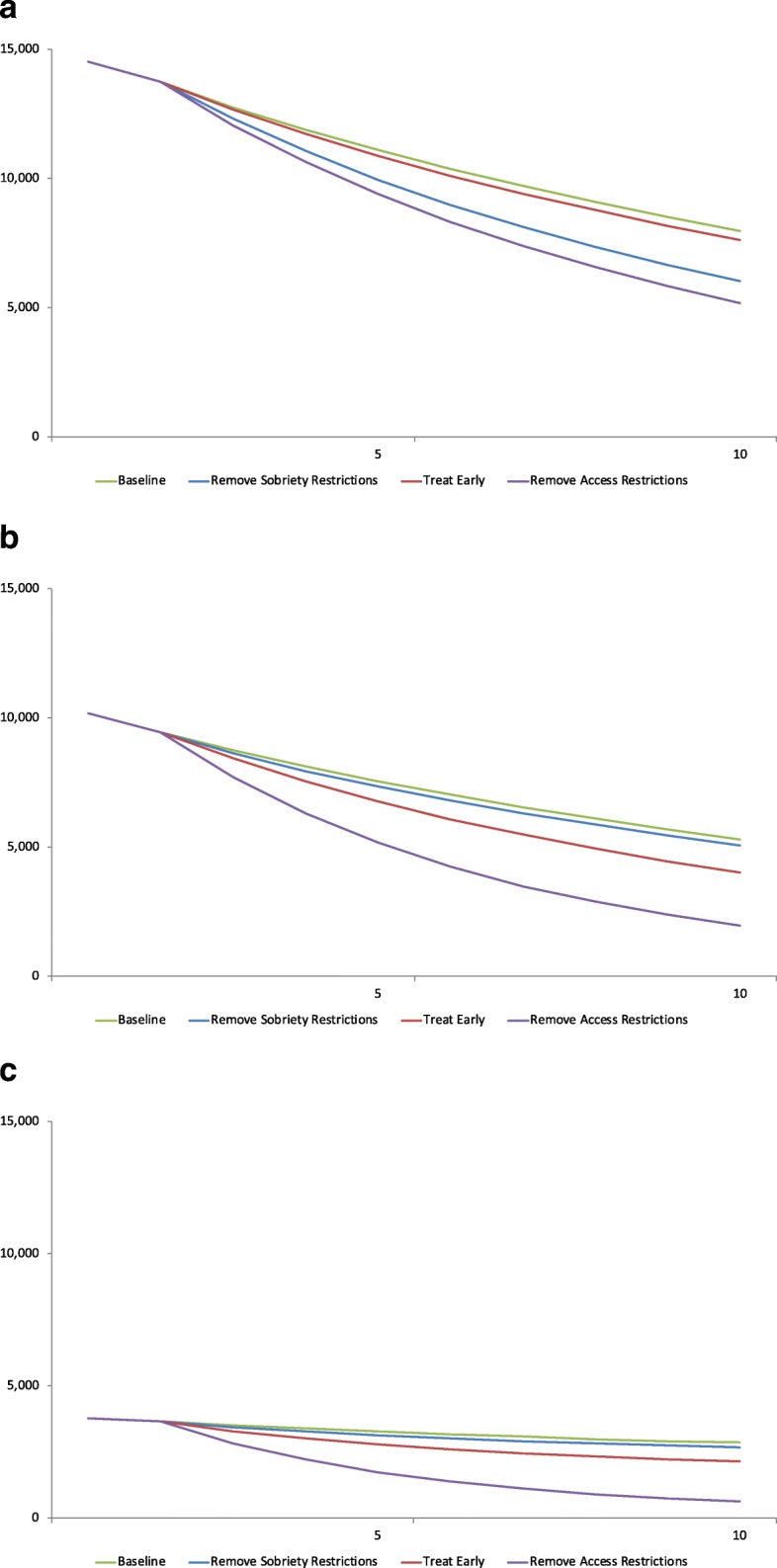
Total infected population per treatment scenario, by state over 10 years. **a** Total infected population, North Carolina. Notes: Baseline treats 6% of patients with Metavir fibrosis score F2 and above, excluding PWIDs. Remove Sobriety Restrictions treats 6% of patients with F2 and above, including PWIDs. Treat Early assumes a 66% diagnosis rate, treating 8% of patients with fibrosis score F0 and above, excluding PWIDs. Remove Access Restrictions treats 8% of patients with F0 and above, including PWIDs. **b** Total infected population, Oregon. Notes: Baseline treats 10% of patients with Metavir fibrosis score F3 and above, excluding PWIDs. Remove Sobriety Restrictions treats 10% of patients with F3 and above, including PWIDs. Treat Early assumes a 66% diagnosis rate, treating 13% of patients with fibrosis score F0 and above, excluding PWIDs. Remove Access Restrictions treats 13% of patients with F0 and above, including PWIDs. **c** Total infected population, Wisconsin. Notes: Baseline treats 18% of patients with Metavir fibrosis score F3 and above, excluding PWIDs. Remove Sobriety Restrictions treats 18% of patients with F3 and above, including PWIDs. Treat Early assumes 66% a diagnosis rate, treating 24% of patients with fibrosis score F0 and above, excluding PWIDs. Remove Access Restrictions treats 24% of patients with F0 and above, including PWIDs

In all three state Medicaid programs, even the most restrictive access scenario (Baseline) resulted in declining HCV-infected populations (Fig. 2). However, each of the alternative treatment access scenarios resulted in larger and more rapid reductions in the HCV-infected population relative to the Baseline scenario. In all states, removing all access restrictions (Remove Access Restrictions) resulted in the largest reduction in the total infected population at year 10 compared to Baseline. In North Carolina (Fig. 2a), the initial HCV-infected population of 14,500 Medicaid enrollees was reduced to 5200 individuals by year 10 with the elimination of all access restrictions, compared to 7600 for Treat Early, 6000 for Remove Sobriety Restrictions, and 8000 for Baseline. Similarly, in Oregon (Fig. 2b), the infected population declined from 10,200 at the start of observation to 2000 individuals after 10 years under the scenario where all access restrictions are removed. By year 10, the population decreased to 4000 in Treat Early, 5000 in Remove Sobriety Restrictions, and 5300 in Baseline. Lastly, Wisconsin (Fig. 2c), which started with the smallest initial HCV prevalent population (*n* = 3800), saw a decline by year 10 to 614 cases in the Remove Access Restrictions scenario, 2100 for Treat Early, 2700 for Remove Sobriety Restrictions, and 2800 for Baseline.

### HCV-related healthcare expenditures

Relative to Baseline, all treatment access policy scenarios initially show an increase in cumulative HCV-related medical and treatment expenditures net Baseline expenditures as larger groups of patients are able to access DAA therapy. As the infected population size decreases, expenditures also decrease, with expenditures under each scenario breaking even with and then decreasing relative to Baseline over the 10-year simulation period (Table 2). We report the number treated per year in the Technical Appendix (Additional file 1: Table S10), and the total number treated over 10 years in Table 2 to contextualize the total spending over the same time period.

**Table 2:** Maximum cumulative medical and treatment expenditures, break-even years, and number treated by treatment scenario, relative to Baseline ($ millions)

	Remove Sobriety Restrictions	Treat Early	Remove Access Restrictions
North Carolina			
Maximum	$19.8	$3.0	$26.0
Year of maximum	2018	2017	2017
Year of break even	2023	2019	2021
Total net of Baseline over 10 years	-$6.8	-$25.7	-$65.9
Total number treated over 10 years	4715	2167	5895
Oregon			
Maximum	$22.6	$2.9	$29.0
Year of maximum	2018	2017	2017
Year of break even	2023	2019	2021
Total net of Baseline over 10 years	$4.9	-$24.9	-$50.4
Total number treated over 10 years	4747	2069	5573
Wisconsin			
Maximum	$13.4	$1.9	$18.2
Year of maximum	2018	2017	2017
Year of break even	2022	2019	2020
Total net of Baseline over 10 years	-$17.2	-$14.4	-$53.8
Total number treated over 10 years	2750	1075	3115

**Fig. 3:**
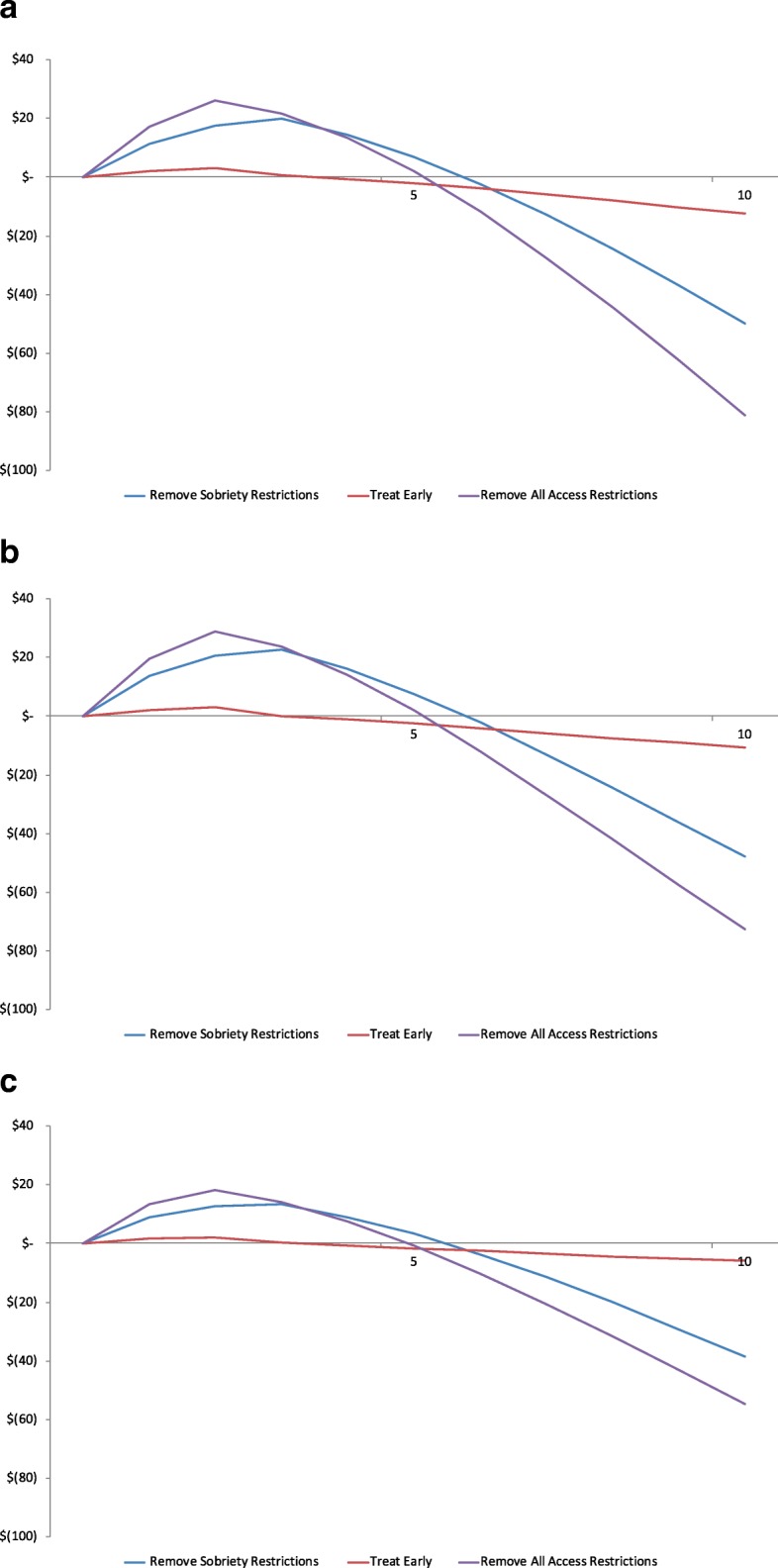
Cumulative medical and treatment expenditures per treatment scenario, relative to Baseline, by state over 10 years ($ millions). **a** Cumulative medical and treatment expenditures, North Carolina. Notes: Baseline treats 6% of patients with Metavir fibrosis score F2 and above, excluding PWIDs. Remove Sobriety Restrictions treats 6% of patients with F2 and above, including PWIDs. Treat Early assumes a 66% diagnosis rate, treating 8% of patients with fibrosis score F0 and above, excluding PWIDs. Remove Access Restrictions treats 8% of patients with F0 and above, including PWIDs. **b** Cumulative medical and treatment expenditures, Oregon. Notes: Baseline treats 10% of patients with Metavir fibrosis score F3 and above, excluding PWIDs. Remove Sobriety Restrictions treats 10% of patients with F3 and above, including PWIDs. Treat Early assumes a 66% diagnosis rate, treating 13% of patients with fibrosis score F0 and above, excluding PWIDs. Remove Access Restrictions treats 13% of patients with F0 and above, including PWIDs. **c** Cumulative medical and treatment expenditures, Wisconsin. Notes: Baseline treats 18% of patients with Metavir fibrosis score F3 and above, excluding PWIDs. Remove Sobriety Restrictions treats 18% of patients with F3 and above, including PWIDs. Treat Early assumes a 66% diagnosis rate, treating 24% of patients with fibrosis score F0 and above, excluding PWIDs. Remove Access Restrictions treats 24% of patients with F0 and above, including PWIDs

In NC (Fig. 3a), OR (Fig. 3b), and WI (Fig. 3c), removing all access restrictions resulted in the greatest reduction in total expenditures net of Baseline over 10 years (-$66 M, -$50 M, and -$54 M, respectively) compared to Remove Sobriety Restrictions (-$7 M, $5 M above Baseline, and -$17 M, respectively) and Treat Early (-$26 M, -$25 M, and -$14 M, respectively). Despite differences in population size and the Baseline scenario, the break-even years for NC, OR, and WI were similar for all three states, with Treat Early offering the earliest breakeven point (2019), but with the least reduction in overall expenditures net of Baseline over the entire 10-year simulation period. Although Remove Access Restrictions results in the highest maximum spending in a given year across all states, it also exhibits the largest reduction in medical expenditures on the whole and allows treatment of the largest number of infected patients (5895 in NC, 5573 in OR, and 3115 in WI vs. Treat Early 2167 in NC, 2069 in OR, and 1075 in WI vs. Remove Sobriety Restrictions 4715 in NC, 4747 in OR, and 2750 in WI). As restrictions for treatment are removed, the pool of patients eligible for treatment grows in absolute numbers, even when the proportion of treated patients remains the same. For example, 8% of patients are treated in both Treat Early and Remove all Access Restrictions in NC, but because PWID are eligible for treatment in Remove All Access Restrictions, the starting population eligible for treatment is larger than in Treat Early, resulting in more patients treated.

### Social value of treatment policies results

**Fig. 4:**
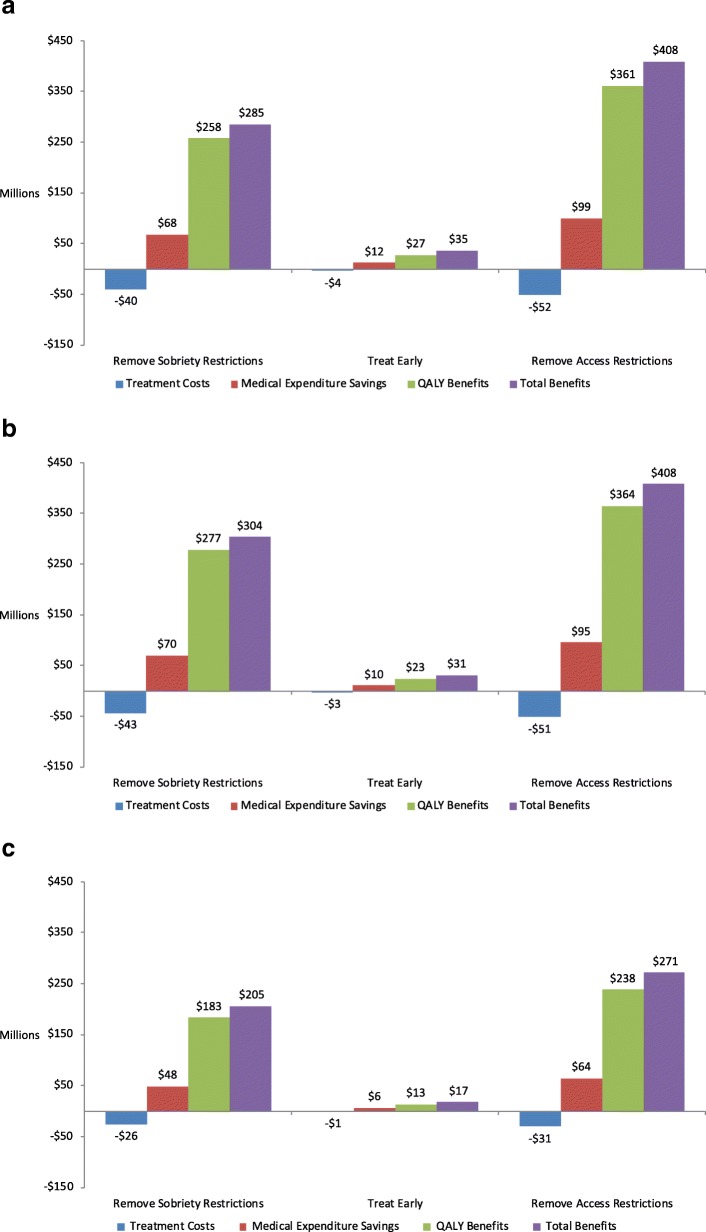
Total social value generated by each treatment scenario, relative to Baseline over 10 years($ millions), 3% discount rate, QALYs valued at $150,000 each. **a** Total social value, North Carolina. Notes: Baseline treats 6% of patients with Metavir fibrosis score F2 and above, excluding PWIDs. Remove Sobriety Restrictions treats 6% of patients with F2 and above, including PWIDs. Treat Early assumes a 66% diagnosis rate, treating 8% of patients with fibrosis score F0 and above, excluding PWIDs. Remove Access Restrictions treats 8% of patients with F0 and above, including PWIDs. **b** Total social value, Oregon. Notes: Baseline treats 10% of patients with Metavir fibrosis score F3 and above, excluding PWIDs. Remove Sobriety Restrictions treats 10% of patients with F3 and above, including PWIDs. Treat Early assumes a 66% diagnosis rate, treating 13% of patients with fibrosis score F0 and above, excluding PWIDs. Remove Access Restrictions treats 13% of patients with F0 and above, including PWIDs. **c** Total social value, Wisconsin. Notes: Baseline treats 18% of patients with Metavir fibrosis score F3 and above, excluding PWIDs. Remove Sobriety Restrictions treats 18% of patients with F3 and above, including PWIDs. Treat Early assumes a 66% diagnosis rate, treating 24% of patients with fibrosis score F0 and above, excluding PWIDs. Remove Access Restrictions treats 24% of patients with F0 and above, including PWIDs

Over the 10-year simulation period, substantial social value, measured as the increase in QALYs net of HCV-related health care expenditures, was generated in each state under each treatment scenario relative to Baseline. In NC (Fig. 4a), the net social value over the 10-year simulation period relative to Baseline was $35 M in Treat Early, $285 M in Remove Sobriety Restrictions, and $408 M in Remove Access Restrictions. Similarly, in OR (Fig. 4b), access to early treatment (Treat Early) generated $31 M in social value, while Remove Sobriety Requirements generated $304 M, and Remove Access Restrictions generated $408 M relative to Baseline. Patterns were also similar for WI (Fig. 4c), although the relative sizes of the benefits were smaller due to the smaller starting population ($17 M, $205 M, and $271 M, respectively). We conducted a sensitivity analysis varying the value of a QALY, and all alternate values used ($50,000, $100,000, and $200,000 per QALY) resulted in positive social value generated, although with larger benefits with increased QALY value (Additional file 1: Table S11). Additional sensitivity analyses were conducted to test the sensitivity of the results to varying the growth rate for the PWID and HIV+/MSM susceptible population (increasing and decreasing the growth rate by 5% for both populations) and changing to an 8-week regimen for eligible populations, rather than a 12-week regimen. The details and results of these sensitivity analyses can also be found in Additional file 1: Tables S12-S14.

### State spending results

Analysis of the impact of access policies on state Medicaid spending showed substantial savings across each of the scenarios relative to Baseline spending. Savings were achieved both with direct state monies as well as with the federal contribution. In NC, total 10-year cumulative state spending decreased by $3-20 M, relative to Baseline, with the greatest savings experienced in the least restrictive access scenario. Similar results were found for OR and WI, with savings ranging from $3-21 M, and $2-18 M, respectively. A concomitant decrease in the federal contribution was also observed with the greatest benefits found in the open access scenario, with savings totaling $99 M combined across all three states.

## Discussion

The availability of effective DAAs for HCV infection has been life-altering for many patients. Prior to the advent of these therapies, treatment was burdensome and toxic, and the cure rate was inconsistent [32]. However, not all patients are able to access these treatments through state Medicaid programs due to a focus on very short-run (1–2 year) budget concerns. However, in keeping with the 10-year time horizon used by the Congressional Budget Office for budget projections [2], we found that all our simulated treatment access policies that expanded access were able to achieve break-even costs compared to Baseline within 4 to 8 years of model start.

The potential benefits of broad access to DAAs in the US was previously estimated by Van Nuys et al. [14]. This research extends those findings with a specific focus on three state Medicaid programs with restrictive access policies. The 2015 state Medicaid policies in NC, OR, and WI restricted treatment to patients with more severe disease and demonstrated sobriety (i.e., no current or recent injection drug use). The impact of more open treatment access policies on the size of the HCV population, HCV medical and treatment expenditures, overall social value, and state spending were modeled over a 10-year simulation period. In all three states, treating all patients with diagnosed HCV regardless of disease severity and sobriety status had the greatest impact on outcomes when compared to other modeled policy scenarios. Not only did removing access barriers substantially reduce the number of patients infected with HCV, it also decreased HCV-related health care expenditures over 10 years, with the initial investment in higher rates of treatment being offset in the first 5–6 years. The overall social value generated from increasing access broadly was substantial. Similar results, although of lesser magnitude, were found with the other treatment access policy scenarios, including treating those at earlier stages of disease and separately removing sobriety requirements. Finally, with the federal government matching a percentage of the spending for each state, the actual burden on states over time is further reduced.

Other recently published studies support broader HCV treatment access policies for state Medicaid programs. Chidi et al. (2016) used a Markov model to compare Medicaid policies to treat patients aged 45–55 with advanced HCV genotype 1 only (restricted access) to policies that treat both early-stage and advanced disease [12]. They assessed the impact of these treatment strategies on CMS as a whole, evaluating downstream clinical and cost effects of restrictive versus full access treatment on Medicare when those patients become eligible. Overall, they found that cases of hepatocellular carcinoma, liver transplants and mortality – all manifestations of severe disease – were substantially reduced with full access compared to the restrictive treatment strategy. Additionally, full access yielded both lower costs and higher QALYs compared to restrictive access. The authors also note that full access treatment is cost-effective and that the budget impact would break even with the restricted access strategy in 9–13 years. While the assumptions around treatment costs differ greatly between Chidi et al. and this research, the direction and magnitude of our findings were similar.

Younossi et al. (2017) also used a Markov model to evaluate economic and clinical outcomes for HCV patients with genotype 1 based on state-specific Medicaid treatment restrictions versus open treatment access over a lifetime horizon [13]. Patients were either: 1) untreated, and only received treatment upon aging into Medicare, or 2) were treated under a Medicaid open access scenario. Similar to Chidi et al., the results showed fewer cases of hepatocellular carcinoma, liver transplants, and cirrhosis, and reduced HCV-related mortality under the open treatment access strategy compared to the treatment restrictions scenario. This translated into an estimated $3.8 billion in overall healthcare cost savings over the lifetimes of the study cohort. While there were key differences between the approach and parameters used in Younossi et al. and the current research, including the time horizon modeled (lifetime versus 10-year), and treatment cost assumptions (annual WAC price increases versus dynamic real-world price reductions to treatment costs throughout model duration), the overall conclusions of the studies were the same: increasing access to DAAs for the Medicaid population ultimately results in lower costs and better outcomes over time.

Our study validates previous work that open access to HCV treatment reduces disease prevalence and is cost-saving in the long-term. We also make a unique case for removing HCV treatment access restrictions by conducting the analysis in three specific state Medicaid programs to compare the impact of access policies. Although several states have removed or reduced treatment access restrictions since 2015 [6, 33], there remain opportunities for other states to allow DAA treatment access to a broader HCV-infected population.

Our study had several limitations. First, the model is populated with literature-based parameters from the US, which may introduce uncertainty and imperfect measures into the analysis. Second, our intention was to model a few states most representative of the population and policies throughout the country, rather than to model the largest number of people infected with HCV. Although we did not model more populous states such as California or New York, we were able to capture variety in Medicaid policies and impacts of interventions that can inform policies in the more populous states. We would expect the more populous states to have larger upfront expenditures but similar break-even patterns as the benefit from reduced transmission would also be greater. Third, this model created artificial boundaries for population mixing, allowing each exposure group to only interact with itself. This does not reflect real-world realities, nor is it likely that a population within a state only remain in that state. However, these choices were deliberate and meant to simplify the assumptions for the model intended to serve as a simulation exercise of a few case examples. Fourth, the model does not incorporate the impact of behavioral changes, such as substance use disorder treatment programs, on disease transmission. Finally, we model only HCV-related health care expenditures. Costs of screening for HCV, costs of diagnostic and eligibility tests such as liver scans and tests for sobriety, and all other medical expenditures are not included in the model as the causality between treatment and the impact on other expenditures would have introduced additional assumptions requiring sufficient evidence. The implications for reducing transmission through treatment and removing sobriety restrictions on screening and diagnosis behavior merits further research.

Medicaid policies that expand screening and treatment for HCV have greater impact on reducing disease prevalence compared with those that expand screening or treatment alone. However, the greatest social value is generated by policies that expand treatment to PWIDs and all patients regardless of disease severity. Federal contributions to state Medicaid programs are also essential to sharing the initial investment from expanded access to treatment. Our findings suggest that states should consider revising their HCV treatment policies to include all individuals infected with HCV in order to achieve significant patient- and population-level benefits.

## Conclusions

While increasing treatment access in Medicaid will raise short-term costs, it will also provide clear benefits by saving money and improving health within a 10-year window. Patients and taxpayers would benefit by considering these gains and viewing HCV treatment policies beyond 1–2 year windows.

## Additional file

Additional file 1: HCV-Medicaid-Social-Value – Appendix-Revised.v2. Short-term budget affordability of hepatitis C treatments for state Medicaid programs – Technical Appendix. The technical appendix contains detailed documentation of the starting model populations, the modeled disease transmission rates, assumptions regarding treatments modeled, and additional parameters such as mortality and transition probabilities for each population modeled. The technical appendix also reports results on the number of patients treated by state and treatment access scenario over 10 years, as well as the results of sensitivity analyses testing QALY value, population growth, and treatment regimen efficiacy and pricing. Finally, the technical appendix provides additional description of the state spending analysis along with the year-and state-specific FMAPs (DOCX 345 kb)

## Abbreviations

CDC: Centers for Disease Control and Prevention
CMS: Centers for Medicare & Medicaid Services
CY: Calendar year
DAA: Direct-acting antiviral
FFS: Fee-for-service
FMAP: Federal Medical Assistance Percentage
FY: Fiscal year
HCV: Hepatitis C
MSM: Men who have sex with men
NC: North Carolina
OR: Oregon
PWID: People who inject drugs
QALY: Quality-adjusted life-year
RNA: Ribonucleic acid
SVR: Sustained virological response
TaP: Transmission and progression
WAC: Wholesale acquisition cost
WI: Wisconsin
