# Supplementary material for: Short-term budget affordability of hepatitis C treatments for state Medicaid programs
Source: BMC Health Serv Res. 2019 Feb 28;19:140. doi: 10.1186/s12913-019-3956-x (PMC6394005; doi:10.1186/s12913-019-3956-x)
Supplement: Supplementary file 1 — HCV-Medicaid-Social-Value – Appendix-Revised.v2. Short-term budget affordability of hepatitis C treatments for state Medicaid programs – Technical Appendix. The technical appendix contains detailed documentation of the starting model populations, the modeled disease transmission rates, assumptions regarding treatments modeled, and additional parameters such as mortality and transition probabilities for each population modeled. The technical appendix also reports results on the number of patients treated by state and treatment access scenario over 10 years, as well as the results of sensitivity analyses testing QALY value, population growth, and treatment regimen efficiacy and pricing. Finally, the technical appendix provides additional description of the state spending analysis along with the year-and state-specific FMAPs (DOCX 345 kb) [file 12913_2019_3956_MOESM1_ESM.docx]

**Short-term budget affordability of hepatitis C treatments for state Medicaid programs**

**Jacquelyn W. Chou, Alison R. Silverstein, Dana P. Goldman**

**Technical Appendix**

**Starting Populations**

We estimated the incidence of HCV at the state level using data from state public health department reports,[1-3] inflating these values by a factor of 12.3 as described in the widely cited Klevens et al. 2014 publication.[4] We converted the scaled incidence for each state into a rate per the estimated size of the state-specific population in each exposure group. State-specific prevalence estimates were calculated in a recent publication by Rosenberg et al.[5] We chose the CID Sensitivity Analysis (RNA+ Method 2) prevalence figures from Rosenberg et al. as they were based on confirmatory HCV ribonucleic acid (RNA) tests, which better account for the subpopulation of patients who would actually be treated compared to the prevalence identified using HCV antibody testing.[6] Other prevalence estimates, such as those from the National Health and Nutrition Examination Survey (NHANES), lack data from confirmatory RNA testing. Starting incidence and prevalence by state can be found in eTable 1.

| Table S1. Starting Incidence and Prevalence Populations by State | | |
| --- | --- | --- |
| **State** | **Incidence** | **Prevalence** |
| North Carolina | 1,390 | 79,500 |
| Oregon | 308 | 55,800 |
| Wisconsin | 722 | 20,700 |
| Incidence inflated with Klevens factor (12.3)  Prevalence estimates are reported with 95% confidence intervals in Rosenberg et al. but the range has not been tested in this model | | |

We began the simulations in each state with the population sizes detailed above. The prevalence in each state was distributed across all disease stages and exposure groups. The incident patients in the PWID and HIV+/MSM exposure groups represent patients in the acute phases of disease at the start of the model. The remaining chronically infected population was distributed across disease stages, genotypes, and exposure groups.[7, 8] We assumed 9% of the At Risk population, 31% of the PWID population, and 18% of the HIV+/MSM population receive treatment and care through Medicaid. We describe the starting infected population by exposure group and disease stage by state in eTable 2 through eTable 4.

| Table S2. Size and distribution of North Carolina model populations at start of simulation | | | | |
| --- | --- | --- | --- | --- |
|  | | **At Risk** | **PWID** | **HIV+/MSM** |
| Uninfected/Susceptible | | 3,162,266 | 186,782 | 12,125 |
| *Genotype 1* | *Acute* | 0 | 364 | 46 |
|  | *F0* | 5,352 | 3,831 | 479 |
|  | *F1* | 11,019 | 7,885 | 986 |
|  | *F2* | 6,926 | 4,957 | 620 |
|  | *F3* | 4,407 | 3,155 | 394 |
|  | *F4* | 1,889 | 1,351 | 169 |
|  | *DC* | 944 | 675 | 84 |
|  | *HCC* | 944 | 675 | 84 |
| *Genotype 2* | *Acute* | 0 | 81 | 10 |
|  | *F0* | 1,189 | 851 | 106 |
|  | *F1* | 2,449 | 1,752 | 219 |
|  | *F2* | 1,539 | 1,102 | 138 |
|  | *F3* | 979 | 701 | 88 |
|  | *F4* | 420 | 300 | 38 |
|  | *DC* | 210 | 150 | 19 |
|  | *HCC* | 210 | 150 | 19 |
| *Genotype 3* | *Acute* | 0 | 61 | 8 |
|  | *F0* | 892 | 638 | 80 |
|  | *F1* | 1,836 | 1,314 | 164 |
|  | *F2* | 1,154 | 826 | 103 |
|  | *F3* | 735 | 526 | 66 |
|  | *F4* | 315 | 225 | 28 |
|  | *DC* | 157 | 113 | 14 |
|  | *HCC* | 157 | 113 | 14 |
| *SOURCES:* Census,[9] Lansky,[10] Williams,[11] Manos,[12] Rosenberg,[5] Hagan,[13] CDC[14] | | | | |

| Table S3. Size and distribution of Oregon model populations at start of simulation | | | | |
| --- | --- | --- | --- | --- |
|  | | **At Risk** | **PWID** | **HIV+/MSM** |
| Uninfected/Susceptible | | 1,335,603 | 66,363 | 3,010 |
| *Genotype 1* | *Acute* | 0 | 256 | 32 |
|  | *F0* | 3,756 | 2,689 | 336 |
|  | *F1* | 7,734 | 5,535 | 692 |
|  | *F2* | 4,861 | 3,479 | 435 |
|  | *F3* | 3,094 | 2,215 | 277 |
|  | *F4* | 1,326 | 948 | 119 |
|  | *DC* | 663 | 474 | 59 |
|  | *HCC* | 663 | 474 | 59 |
| *Genotype 2* | *Acute* | 0 | 57 | 7 |
|  | *F0* | 835 | 597 | 75 |
|  | *F1* | 1,719 | 1,230 | 154 |
|  | *F2* | 1,080 | 773 | 97 |
|  | *F3* | 687 | 492 | 62 |
|  | *F4* | 295 | 211 | 26 |
|  | *DC* | 147 | 105 | 13 |
|  | *HCC* | 147 | 105 | 13 |
| *Genotype 3* | *Acute* | 0 | 43 | 5 |
|  | *F0* | 626 | 448 | 56 |
|  | *F1* | 1,289 | 922 | 115 |
|  | *F2* | 810 | 580 | 72 |
|  | *F3* | 516 | 369 | 46 |
|  | *F4* | 221 | 158 | 20 |
|  | *DC* | 110 | 79 | 10 |
|  | *HCC* | 110 | 79 | 10 |
| *SOURCES:* Census,[9] Lansky,[10] Williams,[11] Manos,[12] Rosenberg,[5] Hagan,[13] CDC[14] | | | | |

| Table S4. Size and distribution of Wisconsin model populations at start of simulation | | | | |
| --- | --- | --- | --- | --- |
|  | | **At Risk** | **PWID** | **HIV+/MSM** |
| Uninfected/Susceptible | | 1,945,900 | 117,833 | 2,965 |
| *Genotype 1* | *Acute* | 0 | 95 | 12 |
|  | *F0* | 1,394 | 997 | 125 |
|  | *F1* | 2,869 | 2,053 | 257 |
|  | *F2* | 1,803 | 1,291 | 161 |
|  | *F3* | 1,148 | 822 | 103 |
|  | *F4* | 492 | 352 | 44 |
|  | *DC* | 246 | 176 | 22 |
|  | *HCC* | 246 | 176 | 22 |
| *Genotype 2* | *Acute* | 0 | 21 | 3 |
|  | *F0* | 310 | 222 | 28 |
|  | *F1* | 638 | 456 | 57 |
|  | *F2* | 401 | 287 | 36 |
|  | *F3* | 255 | 183 | 23 |
|  | *F4* | 109 | 78 | 10 |
|  | *DC* | 55 | 39 | 5 |
|  | *HCC* | 55 | 39 | 5 |
| *Genotype 3* | *Acute* | 0 | 16 | 2 |
|  | *F0* | 232 | 166 | 21 |
|  | *F1* | 478 | 342 | 43 |
|  | *F2* | 301 | 215 | 27 |
|  | *F3* | 191 | 137 | 17 |
|  | *F4* | 82 | 59 | 7 |
|  | *DC* | 41 | 29 | 4 |
|  | *HCC* | 41 | 29 | 4 |
| *SOURCES:* Census,[9] Lansky,[10] Williams,[11] Manos,[12] Rosenberg,[5] Hagan,[13] CDC[14] | | | | |

## Transmission Function

In the PWID and HIV+/MSM exposure groups, for each genotype, the rate at which individuals are infected is modeled dynamically as a function of the number in the exposure group who are currently infected with the given genotype. We assume no on-going transmission for the At Risk exposure group. Additional details on the transmission calculations can be found in Van Nuys 2015.[15] The incidence rates and proportionality constants *K* used in this state Medicaid-adapted model are available in eTable 5.

| Table S5. Starting Annual Incidence Rates and Values of K | | |
| --- | --- | --- |
|  | **Annual Incidence Rate** | **Calculated *K*** |
| North Carolina | | |
| *At Risk* | | |
| Genotype 1 | 0 | 0 |
| Genotype 2 | 0 | 0 |
| Genotype 3 | 0 | 0 |
| *PWID* | | |
| Genotype 1 | 0.004 | 0.04 |
| Genotype 2 | 0.001 | 0.04 |
| Genotype 3 | 0.0007 | 0.04 |
| *HIV+/MSM* | | |
| Genotype 1 | 0.02 | 0.09 |
| Genotype 2 | 0.003 | 0.09 |
| Genotype 3 | 0.003 | 0.09 |
| Oregon | | |
| *At Risk* |  |  |
| Genotype 1 | 0 | 0 |
| Genotype 2 | 0 | 0 |
| Genotype 3 | 0 | 0 |
| *PWID* |  |  |
| Genotype 1 | 0.003 | 0.01 |
| Genotype 2 | 0.0006 | 0.01 |
| Genotype 3 | 0.0005 | 0.01 |
| *HIV+/MSM* |  |  |
| Genotype 1 | 0.01 | 0.04 |
| Genotype 2 | 0.003 | 0.04 |
| Genotype 3 | 0.002 | 0.04 |
| Wisconsin | | |
| *At Risk* |  |  |
| Genotype 1 | 0 | 0 |
| Genotype 2 | 0 | 0 |
| Genotype 3 | 0 | 0 |
| *PWID* |  |  |
| Genotype 1 | 0.004 | 0.08 |
| Genotype 2 | 0.0008 | 0.08 |
| Genotype 3 | 0.0006 | 0.08 |
| *HIV+/MSM* |  |  |
| Genotype 1 | 0.03 | 0.18 |
| Genotype 2 | 0.007 | 0.18 |
| Genotype 3 | 0.006 | 0.18 |
| *SOURCES:* North Carolina Division of Public Health,[1] Oregon Health Authority,[2] Wisconsin Department of Health Services,[3] and authors’ calculations. | | |

## Treatment Assumptions

We modeled four treatment policy scenarios in North Carolina (NC), Oregon (OR), and Wisconsin (WI) that differ in exposure groups, disease severity, and proportion of diagnosed patients treated. All treatment scenarios assumed treatment with direct-acting antivirals (DAAs), specifically sofosbuvir + velpatasvir. The Baseline scenario in each state was based on Medicaid criteria for HCV treatment in 2015.

In NC, OR, and WI, the Remove Sobriety Restrictions scenario expanded treatment to PWIDs, as they were not included in criteria for treatment in 2015, and maintained the same disease severity criteria and proportion of patients treated. The Treat Early scenario expanded the Baseline scenario disease severity criteria for treatment to F0+ and simulated a 66% diagnosis rate; PWIDs were not treated. The Remove Access Restrictions scenario built upon Treat Early and allowed PWIDs to be treated. The Baseline and all alternative treatment policy scenarios are outlined in eTable 6.

A weighted sustained virological response (SVR) was calculated in order to account for the differences in SVR between treatment-naïve and treatment-experienced patients observed in clinical trials of sofosbuvir + velpatasvir. This weighted SVR was based on 80% of the population never receiving prior treatment and is reported in eTable 6.

All scenarios treated a constant proportion of the infected population in each model cycle. We used a baseline 50% diagnosis rate, consistent with estimates in the US.[6, 16, 17] After the first model cycle, a large proportion of patients move from the prevalent to the susceptible population upon being cured. In each following cycle, we maintained the same diagnosis rate and the same proportion of infected patients were treated.

The wholesale acquisition cost (WAC) of a 12-week course of treatment with sofosbuvir + velpatasvir is $74,760. We calculated a 43% discount to Medicaid, such that the treatment costs modeled are $42,610. The DAAs modeled are currently protected under patent. We modeled two pricing trajectories to examine the effect of actual competition in the market. In years 1 and 2, we implemented a 46% discount from the starting treatment cost to Medicaid. In year 3, the price declines to $10,000 per bottle (3 bottles per 12-week regimen) in treatment access scenarios that expand access to PWIDs (Remove Sobriety Restrictions and Remove Access Restrictions). In year 4, we model the impact to pricing of a competitor product entering the market, reducing the price to $20,300 in all scenarios. Patent expiry would occur in year 15, so is not captured in the pricing dynamics of our model as we simulate outcomes up through 10 years. Patent expiry would be estimated to reduce treatment costs to 79% of their year 1 value in year 15, which is assumed to be the marginal cost of producing the drugs.[18] Regimen drugs, duration, efficacy, and treatment costs are detailed in eTable 6.

| Table S6. Regimens, Duration, and Efficacy for Four Treatment Scenarios Modeled | | | | | | | | | | |
| --- | --- | --- | --- | --- | --- | --- | --- | --- | --- | --- |
|  | | **Baseline** | | | | | **Expand Screening** | | | |
|  | | **Baseline** | | | **Remove Sobriety Restrictions** | | **Treat Early** | | **Remove Access Restrictions** | |
| North Carolina | | F2+, Treat 6%, no PWIDs | | | Treat PWIDs, F2+, Treat 6% | | Treat 8%, F0+, no PWIDs | | Treat PWIDs, Treat 8%, F0+ | |
| Oregon | | F3+, Treat 10%, no PWIDs | | | Treat PWIDs, F3+, Treat 10% | | Treat 13%, F0+, no PWIDs | | Treat PWIDs, Treat 13%, F0+ | |
| Wisconsin | | F3+, Treat 18%, no PWIDs | | | Treat PWIDs, F3+, Treat 18% | | Treat 24%, F0+, no PWIDs | | Treat PWIDs, Treat 24%, F0+ | |
| Drugs used | | sofosbuvir + velpatasvir 12 weeks | | | | | | | | |
| Treatment cost | | $43,000 | | | | | | | | |
| SVR by disease stage | | **F0-F3** | | **F4, DC, HCC** | **F0-F3** | **F4, DC, HCC** | **F0-F3** | **F4, DC, HCC** | **F0-F3** | **F4, DC, HCC** |
| Genotype 1 | At Risk | 0.98 | 1.00 | | Same as Baseline | | | | | |
|  | PWID | 0.98 | 1.00 | | Same as Baseline | | | | | |
|  | HIV+/MSM | 0.96 | 0.96 | | Same as Baseline | | | | | |
| Genotype 2 | At Risk | 0.99 | 1.00 | | Same as Baseline | | | | | |
|  | PWID | 0.99 | 1.00 | | Same as Baseline | | | | | |
|  | HIV+/MSM | 1.00 | 1.00 | | Same as Baseline | | | | | |
| Genotype 3 | At Risk | 0.97 | 0.92 | | Same as Baseline | | | | | |
|  | PWID | 0.97 | 0.92 | | Same as Baseline | | | | | |
|  | HIV+/MSM | 0.92 | 0.91 | | Same as Baseline | | | | | |

# Model Parameters

Disease stage progression and distribution parameters were estimated from the literature, specifically for each exposure group and genotype, when available. Disease stage progression varied by exposure group.

The At Risk exposure group was defined to be US residents born between 1945 and 1965, with population size taken from the US Census.[9] This exposure group contains the largest number of HCV-infected patients, although the cohort shrinks as the simulation progresses. The mortality rate for this group increases at 8% per year as this is a closed cohort aging over time.[19] Model parameters for the At Risk exposure cohort and their sources are provided in eTable 7.

The PWID exposure group was assumed to be ages 13 years and older, comprising the majority of new infections in all three states due to the higher modeled transmission rate.[10] The PWID population has the highest mortality rate of the three exposure groups for both the infected and uninfected populations.[20] Model parameters for the PWID cohort are listed in eTable 8.

The HIV+/MSM exposure group is characterized by co-infection with HIV, which affects the progression of HCV,[21] and in our analysis is principally composed of men who have sex with men. This population was assumed to be male US residents ages 13 years and older with diagnosed and undiagnosed HIV infection due to male-to-male sexual contact.[22] Model parameters for the HIV+/MSM exposure group and their sources are provided in eTable 9.

| Table S7. Model Parameters for the At Risk Exposure Group | | | |
| --- | --- | --- | --- |
|  | **Genotype 1** | **Genotype 2** | **Genotype 3** |
| Annual Mortality Rate | | | |
| Susceptible (Background) | 0.006[9] | | |
| Acute, F0-F2 | 0.01[23, 24] | 0.01[23, 24] | 0.01[23, 24] |
| F3-F4 | 0.05[8, 23, 24] | 0.05[8, 23, 24] | 0.05[8, 23, 24] |
| DC | 0.14[25] | | |
| HCC | 0.43[25] | | |
| DC Transplant, HCC Transplant^a^ | 0.17[26] | | |
| DC Post-Transplant^b^ | 0.10[25] | | |
| HCC Post-Transplant^b^ | 0.32[25] | | |
| Annual Background Mortality Growth Rate | 0.08[9, 19] | | |
|  | | | |
| Annual Transition Probability | | | |
| Acute → Spontaneous Clearance | 0.18[27] | | |
| F0 → F1 | 0.08[28] | | |
| F1→ F2 | 0.10[28] | | |
| F2 → F3 | 0.11[28] | | |
| F3 → F4 | 0.13[28] | | |
| F3 → HCC | 0.008[25, 29] | | 0.01[25, 30] |
| F4 → DC | 0.04[25, 31] | 0.03[25, 30] | 0.05[25, 30] |
| F4 → HCC | 0.03[25, 31] | 0.01[25, 30] | 0.05[25, 30] |
| DC → HCC | 0.03[25, 29] | 0.01[25, 30] | 0.05[25, 30] |
| DC → Transplant | 0.03[32] | | |
| HCC → Transplant | 0.10[32] | | |
| F3 Cure → F4 Cure | 0.04[33] | | |
| F3 Cure → HCC Cure | 0.003[33] | | |
| F4 Cure → DC Cure | 0.10[33] | | |
| F4 Cure → HCC Cure | 0.009[33] | | |
| DC Cure → HCC Cure | 0.007[33] | | |
|  | | | |
| QALY Weights | | | |
| Susceptible | 0.86[32, 34] | | |
| Acute | 0.79 Assumption | | |
| F0-F3, F0-F3 Cure | 0.79[32, 35] | | |
| F4, F4 Cure | 0.76[32, 36] | | |
| DC, DC Cure | 0.69[32, 36] | | |
| HCC, HCC Cure | 0.67[32, 36] | | |
| DC Transplant^a^, HCC Transplant | 0.50[32, 37] | | |
| DC Post-Transplant^b^, HCC Post-Transplant^b^ | 0.71[35, 38] | | |
|  | | | |
| Annual Medical Expenditures (inflated to 2015 USD) | | | |
| Susceptible | $5,905.92[39] | | |
| Acute, F0, F1, F2, F3 | $13,796.26[40] | | |
| F0 Fail, F1 Fail, F2 Fail, F3 Fail | $8,967.57[40, 41] | | |
| F4 | $16,365.02[40] | | |
| F4 Fail | $12,437.42[40, 41] | | |
| DC | $45,699.23[40] | | |
| DC Fail, DC Cure | $31,989.46[40, 41] | | |
| DC Transplant^a^, DC Post-Transplant | $131,488.82[40] | | |
| HCC | $101,390.28[40] | | |
| HCC Fail, HCC Cure | $70,973.20[40, 41] | | |
| HCC Transplant^a^ | $131,488.82[40] | | |
| *NOTE ^a^*Year of liver transplant; ^b^All subsequent years after liver transplant. All disease states are based on the Metavir fibrosis scores.[42] | | | |

| Table S8. Model Parameters for the PWID Exposure Group | | | |
| --- | --- | --- | --- |
|  | **Genotype 1** | **Genotype 2** | **Genotype 3** |
| Annual Mortality Rate | | | |
| Susceptible | 0.03[20] | | |
| Acute, F0-F2 | 0.06[20, 23, 24] | 0.05[20, 23, 24] | 0.06[20, 23, 24] |
| F3 | 0.10[8, 20, 23, 24] | 0.20[8, 20, 23, 24] | 0.24[8, 20, 23, 24] |
| F4 | 0.34[8, 20, 23, 24] | 0.20[8, 20, 23, 24] | 0.24[8, 20, 23, 24] |
| DC, DC Transplant^a^ | 0.14[20, 24, 25] | | |
| HCC, HCC Transplant^a^ | 0.43[20, 24, 25] | | |
| DC Post-Transplant^b^ | 0.10[20, 24, 25] | | |
| HCC Post-Transplant^b^ | 0.32[20, 24, 25] | | |
|  | | | |
| Annual Transition Probability | | | |
| Acute → Spontaneous Clearance | 0.24[43] | | |
| F0 → F1 | 0.12[28] | | |
| F1→ F2 | 0.09[28] | | |
| F2 → F3 | 0.09[28] | | |
| F3 → F4 | 0.13[28] | | |
| F3 → HCC | 0.008[25, 29] | | 0.01[25, 30] |
| F4 → DC | 0.04[25, 31] | 0.03[25, 30] | 0.05[25, 30] |
| F4 → HCC | 0.03[25, 31] | 0.014[25, 30] | 0.05[25, 30] |
| DC → HCC | 0.03[25, 29] | 0.014[25, 30] | 0.05[25, 30] |
| DC → Transplant | 0.031[32] | | |
| HCC → Transplant | 0.10[32] | | |
| F3 Cure → F4 Cure | 0.04[33] | | |
| F3 Cure → HCC Cure | 0.003[33] | | |
| F4 Cure → DC Cure | 0.01[33] | | |
| F4 Cure → HCC Cure | 0.009[33] | | |
| DC Cure → HCC Cure | 0.007[33] | | |
|  | | | |
| QALY Weights | Same as for At Risk exposure group. See eTable 7 for values. | | |
|  | | | |
| Annual Medical Expenditures | Same as for At Risk exposure group. See eTable 7 for values. | | |
| *NOTE ^a^*Year of liver transplant; ^b^All subsequent years after liver transplant. All disease states are based on the Metavir fibrosis scores.[42] | | | |

| Table S9. Model Parameters for the HIV+/MSM Exposure Group | | | |
| --- | --- | --- | --- |
|  | **Genotype 1** | **Genotype 2** | **Genotype 3** |
| Annual Mortality Rate | | | |
| Susceptible | 0.0001[44] | | |
| Acute, F0-F2 | 0.0002[23, 24, 44, 45] | 0.0002 [23, 24, 44, 45] | 0.0002[23, 24, 44, 45] |
| F3 | 0.0009[8, 23, 24, 44, 45] | 0.0008[8, 23, 24, 44, 45] | 0.0009[8, 23, 24, 44, 45] |
| F4 | 0.0009[8, 23, 24, 44, 45] | 0.0008[8, 23, 24, 44, 45] | 0.0009[8, 23, 24, 44, 45] |
| DC, DC Transplant^a^ | 0.14[20, 24, 25, 44] | | |
| HCC, HCC Transplant^a^ | 0.43[20, 24, 25, 44] | | |
| DC Post-Transplant^b^ | 0.10[20, 24, 25, 44] | | |
| HCC Post-Transplant^b^ | 0.32[20, 24, 25, 44] | | |
|  | | | |
| Annual Transition Probability | | | |
| Acute → Spontaneous Clearance | 0.15[43] | | |
| F0 → F1 | 0.12[21] | | |
| F1→ F2 | 0.12[21] | | |
| F2 → F3 | 0.12[21] | | |
| F3 → F4 | 0.12[21] | | |
| F3 → HCC | 0.02[25, 29, 38] | | 0.03[25, 30] |
| F4 → DC | 0.08[25, 31, 38] | 0.05[25, 30] | 0.10[25, 30] |
| F4 → HCC | 0.05[25, 31, 38] | 0.03[25, 30] | 0.09[25, 30] |
| DC → HCC | 0.05[25, 31, 38] | 0.03[25, 30] | 0.09[25, 30] |
| DC → Transplant | 0.0[38, 46] | | |
| HCC → Transplant | 0.0[38, 46] | | |
| F3 Cure → F4 Cure | 0.03[33] | | |
| F3 Cure → HCC Cure | 0.006[33] | | |
| F4 Cure → DC Cure | 0.02[33] | | |
| F4 Cure → HCC Cure | 0.020[33] | | |
| DC Cure → HCC Cure | 0.01[33] | | |
|  | | | |
| QALY Weights | | | |
| Susceptible | 0.87[25, 34, 46-50] | | |
| Acute | 0.81 Assumption | | |
| F0-F3, F0-F3 Cure | 0.81[25, 34, 46-50] | | |
| F4, F4 Cure | 0.68[25, 34, 46-50] | | |
| DC, DC Cure | 0.48[25, 34, 46-50] | | |
| HCC, HCC Cure | 0.23[25, 34, 46-50] | | |
| DC Transplant^a^, HCC Transplant | 0.81[25, 34, 46-50] | | |
| DC Post-Transplant^b^, HCC Post-Transplant^b^ | 0.81[25, 34, 46-50] | | |
|  | | | |
| Annual Medical Expenditures | Same as for At Risk exposure group. See eTable 7 for values. | | |
| *NOTE ^a^*Year of liver transplant; ^b^All subsequent years after liver transplant. All disease states are based on the Metavir fibrosis scores.[42] | | | |

## Number of Patients Treated

In order to understand the impact of treatment eligibility policy changes on the size of the population treated, we estimated the number of patients treated each model cycle and in total over the 10-year modeled period by state and treatment scenario. In NC, more patients were treated under the Remove Sobriety Restrictions scenario compared to Treat Early, although Remove Access Restrictions treated more than 2.5 times the number of patients treated in Remove Sobriety Restrictions. This is likely because at baseline, NC allowed access to treatment at F2; earlier than in OR or in WI. In OR and WI, Remove Access Restrictions consistently treated the most patients, followed by Treat Early and then Remove Sobriety Restrictions. These results reflect the increase in populations of patients eligible for treatment in each state under each scenario. For example, allowing treatment of PWID only under Remove Sobriety Restrictions expands the eligible population more than allowing patients with less severe disease Treat Early in NC as NC was already allowing treatment at F2, where the reverse is true in OR and WI, since access had been limited to advanced disease. Additionally, the reduction in number treated across all states and scenarios each year over the course of the model reflects the reduced number of infected individuals due to disease treatment. Complete results are found in eTable 10.

| **Table S10. Number treated each year by state and treatment access scenario** | | | |
| --- | --- | --- | --- |
| **Year** | Remove Sobriety Restrictions | Treat Early | Remove Access Restrictions |
| North Carolina | | | |
| 1 | - | - | - |
| 2 | 785 | 355 | 1,047 |
| 3 | 691 | 317 | 902 |
| 4 | 619 | 286 | 792 |
| 5 | 557 | 258 | 699 |
| 6 | 502 | 232 | 617 |
| 7 | 452 | 209 | 546 |
| 8 | 408 | 188 | 484 |
| 9 | 368 | 169 | 428 |
| 10 | 333 | 152 | 380 |
| Total over 10 years | 4,715 | 2,167 | 5,895 |
| Oregon | | | |
| 1 | - | - | - |
| 2 | 953 | 416 | 1,225 |
| 3 | 800 | 350 | 995 |
| 4 | 672 | 295 | 810 |
| 5 | 567 | 249 | 661 |
| 6 | 478 | 209 | 540 |
| 7 | 404 | 176 | 442 |
| 8 | 341 | 148 | 362 |
| 9 | 288 | 123 | 296 |
| 10 | 243 | 103 | 242 |
| Total over 10 years | 4,747 | 2,069 | 5,573 |
| Wisconsin | | | |
| 1 | - | - | - |
| 2 | 614 | 278 | 818 |
| 3 | 472 | 205 | 584 |
| 4 | 385 | 156 | 447 |
| 5 | 319 | 121 | 350 |
| 6 | 265 | 94 | 275 |
| 7 | 221 | 74 | 217 |
| 8 | 186 | 59 | 173 |
| 9 | 156 | 48 | 139 |
| 10 | 132 | 40 | 112 |
| Total over 10 years | 2,750 | 1,075 | 3,115 |

## QALY Value Sensitivity Analysis

We tested the sensitivity of the social value results to the value of a QALY at $200,000, $100,000, and $50,000, compared to the base case at $150,000 per QALY for each treatment access scenario in all states. At all QALY values, positive social value was generated over 10 years. The total benefits, or social value, (the summation of treatment costs, medical expenditure savings, and QALY benefits) are presented in eTable 11. Negative treatment costs indicate additional spending, while medical expenditure savings indicate fewer medical expenditures, positively contributing to total value generated, relative to Baseline. Negative total benefits relative to base case indicate fewer benefits as a result from lower valued QALYs.

| **Table S11. Sensitivity analysis of costs and benefits with different QALY values, by state and treatment access scenario over 10 years, relative to Baseline ($ millions, cumulative)** | | | |
| --- | --- | --- | --- |
|  | Remove Sobriety Restrictions | Treat Early | Remove All Access Restrictions |
| North Carolina | | | |
| QALY = $150K (Base case) | | | |
| Treatment Costs | -$40.3 | -$4.0 | -$51.5 |
| Medical Expenditure Savings | $67.7 | $12.3 | $99.1 |
| QALY Benefits | $257.8 | $27.2 | $360.6 |
| Total Benefits | $285.2 | $35.5 | $408.2 |
| Total Benefits, QALY = $200K  (difference from base case) | $371.1  ($85.9) | $44.5  ($9.0) | $528.4  ($120.2) |
| Total Benefits, QALY = $100K  (difference from base case) | $199.2  (-$86.0) | $26.4  (-$9.1) | $288.0  (-$120.2) |
| Total Benefits, QALY = $50K  (difference from base case) | $113.3  (-$171.9) | $17.3  (-$18.2) | $167.8  (-$240.4) |
| Oregon | | | |
| QALY = $150K (Base case) | | | |
| Treatment Costs | -$43.3 | -$2.7 | -$51.1 |
| Medical Expenditure Savings | $69.6 | $10.0 | $94.7 |
| QALY Benefits | $277.3 | $23.2 | $364.3 |
| Total Benefits | $303.6 | $30.5 | $407.9 |
| Total Benefits, QALY = $200K  (difference from base case) | $396.1  ($92.5) | $38.3  ($7.8) | $529.4  ($121.5) |
| Total Benefits, QALY = $100K  (difference from base case) | $211.2  (-$92.4) | $22.8  (-$7.7) | $286.5  (-$121.4) |
| Total Benefits, QALY = $50K  (difference from base case) | $118.8  (-$184.8) | $15.0  (-$15.5) | $165.1  (-$242.8) |
| Wisconsin | | | |
| QALY = $150K (Base case) | | | |
| Treatment Costs | -$26.3 | -$1.4 | -$30.6 |
| Medical Expenditure Savings | $48.1 | $5.6 | $63.7 |
| QALY Benefits | $183.3 | $13.0 | $238.3 |
| Total Benefits | $205.1 | $17.2 | $271.4 |
| Total Benefits, QALY = $200K  (difference from base case) | $266.3  ($61.2) | $21.5  ($4.3) | $350.9  ($79.5) |
| Total Benefits, QALY = $100K  (difference from base case) | $144.1  (-$61.0) | $12.8  (-$4.4) | $192.0  (-$79.5) |
| Total Benefits, QALY = $50K  (difference from base case) | $83.0  (-$122.1) | $8.5  (-$8.7) | $112.6  (-$158.8) |

## Population Growth Sensitivity Analysis

We also tested the sensitivity of results to the growth rates of the PWID and HIV+/MSM populations throughout the simulation for each treatment access scenario in all states, testing a 5% annual growth rate increase and a 5% annual growth rate decrease. We report the results of both instances alongside the base case with a 0% growth rate for the break-even years, number treated over 10 years, and total benefits over 10 years in eTable 12. Results were stable with minimal changes seen in the Treat Early and Remove Access Restrictions scenarios. The results for Remove Sobriety Restrictions were more sensitive to the PWID growth rate, but results were not substantially changed. Because the HIV+/MSM population is a small population relative to the other two exposure groups, changing the growth rate of that cohort had minimal impact on outcomes. However, changing the PWID growth rate more substantially altered the number of patients that were treated in both the Remove Sobriety Restrictions scenario and the Remove Access Restrictions scenario.

| **Table S12. Effect of varying the growth rate for the PWID and HIV+/MSM susceptible population on cumulative medical and treatment expenditure break-even years, number treated, and total benefits relative to Baseline, by state and treatment access scenario (QALY = $150K; $ millions)** | | | | | | | | | |
| --- | --- | --- | --- | --- | --- | --- | --- | --- | --- |
|  | Remove Sobriety Restrictions | | | Treat Early | | | Remove All Access Restrictions | | |
|  | **Base case** | **+5% growth** | **-5% growth** | **Base case** | **+5% growth** | **-5% growth** | **Base case** | **+5% growth** | **-5% growth** |
| North Carolina | | | | | | | | | |
| Year of Break-even | 2023 | 2021 | 2021 | 2019 | 2019 | 2019 | 2021 | 2021 | 2021 |
| Number Treated | 4,715 | 4,719 | 4,709 | 2,167 | 2,169 | 2,164 | 5,895 | 5,900 | 5,888 |
| Total Benefits | $285.2 | $287.5 | $284.1 | $35.5 | $35.6 | $35.3 | $408.2 | $407.9 | $406.7 |
| Oregon | | | | | | | | | |
| Year of Break-even | 2023 | 2021 | 2021 | 2019 | 2019 | 2019 | 2021 | 2021 | 2021 |
| Number Treated | 4,747 | 4,750 | 4,744 | 2,069 | 2,071 | 2,068 | 5,573 | 5,576 | 5,570 |
| Total Benefits | $303.6 | $304.1 | $303.1 | $30.5 | $30.6 | $30.5 | $407.9 | $408.5 | $407.2 |
| Wisconsin | | | | | | | | | |
| Year of Break-even | 2022 | 2021 | 2021 | 2019 | 2019 | 2019 | 2020 | 2020 | 2021 |
| Number Treated | 2,750 | 2,754 | 2,749 | 1,075 | 1,078 | 1,075 | 3,115 | 3,119 | 3,134 |
| Total Benefits | $205.1 | $205.7 | $195.4 | $17.2 | $17.3 | $17.1 | $271.4 | $272.2 | $259.1 |

## Treatment Regimen Efficacy and Pricing Sensitivity Analysis

In order to examine the impact of alternative real-world treatment regimens, we sought to model the impacts of an 8-week regimen of glecaprevir + pibrentasvir for eligible populations. Currently, patients F0-F3 across all genotypes are eligible for an 8-week treatment regimen. To determine the impact of the newer 8-week treatment regimen, we assumed patients F0-F3 in all exposure groups received the 8-week regimen and assumed treatment with the 12-week regimen in the base case (both in terms of SVR and treatment costs) for modeled populations who were ineligible for current 8-week treatment. As with the 12-week regimen, a weighted SVR was calculated in order to account for the differences in SVR between treatment-naïve and treatment-experienced patients observed in clinical trials of 8-week glecaprevir + pibrentasvir treatment. This weighted SVR was based on 80% of the population never receiving prior treatment and 96% of those patients being eligible for an 8-week regimen. We calculated an SVR for the 8-week regimen where data were available and assumed SVR from the 12-week regimen where 8-week data were unavailable. We calculate these SVR based on the clinical trial results cited in the table below. The efficacy and pricing for the 8-week regimen are available in eTable 13. Cost of treatment for eligible populations is based on WAC of an 8-week course of treatment with glecaprevir + pibrentasvir at $26,400. Incorporating cost of treatment with 12-week sofosbuvir + velpatasvir for populations not eligible for 8-week glecaprevir + pibrentasvir, we calculated a 43% discount to Medicaid, such that the treatment costs modeled are $20,624. As in the base case, we assume patent expiry would occur in year 15; thus is not captured in the pricing dynamics of our model as we simulate outcomes up through 10 years. Patent expiry would be estimated to reduce treatment costs to 79% of their year 1 value in year 15, which is assumed to be the marginal cost of producing the drugs.[18]

The breakeven year and number treated remain largely unchanged with the 8-week treatment regimen. For each treatment access scenario, the total benefits (treatment costs, medical expenditure savings, and QALY benefits) are calculated relative to the Baseline access restrictions modeled in each state. We see greater value generated with the 8-week treatment regimen under Remove Sobriety Restrictions and Remove all Access Restrictions compared to the 12-week regimen in all states. Treat Early generates approximately the same or slightly less value with the 8-week treatment regimen compared to the 12-week regimen. Treat Early total benefits are not consistently lower in the 8-week treatment scenario as the Baseline is also altered to enable access to 8-weetreatment costs and SVRs. Overall, results of the 8-week treatment scenarios are not substantially different from our base case. Results of the 8-week regimen sensitivity analysis are found in eTable 14.

| **Table S13. Treatment costs and efficacy for 8-week treatment regimen** | | |
| --- | --- | --- |
|  | |  |
| Disease stage | | **F0-F3** |
| Treatment cost (WAC) | | $26,400 |
| Genotype 1 | At Risk | 0.99[51] |
|  | PWID | 0.99[51] |
|  | HIV+/MSM | 1.00[51, 52] |
| Genotype 2 | At Risk | 0.98[53] |
|  | PWID | 0.98[53] |
|  | HIV+/MSM | 1.00[52] |
| Genotype 3 | At Risk | 0.94[51] |
|  | PWID | 0.94[51] |
|  | HIV+/MSM | 0.98[52] |

| **Table S14. Effect of changing to an 8-week treatment regimen for eligible populations on cumulative medical and treatment expenditure break-even years, number treated, and total benefits relative to Baseline, by state and treatment access scenario (QALY = $150K; $ millions)** | | | | | | |
| --- | --- | --- | --- | --- | --- | --- |
|  | Remove Sobriety Restrictions | | Treat Early | | Remove All Access Restrictions | |
|  | **Base case** | **8-week Treatment** | **Base case** | **8-week Treatment** | **Base case** | **8-week Treatment** |
| North Carolina | | | | | | |
| Year of Break-even | 2023 | 2020 | 2019 | 2019 | 2021 | 2020 |
| Number Treated | 4,715 | 4,715 | 2,167 | 2,167 | 5,895 | 5,894 |
| Total Benefits | $285.2 | $295.6 | $35.5 | $35.0 | $408.2 | $417.7 |
| Oregon | | | | | | |
| Year of Break-even | 2023 | 2020 | 2019 | 2019 | 2021 | 2019 |
| Number Treated | 4,747 | 4,747 | 2,069 | 2,069 | 5,573 | 5,573 |
| Total Benefits | $303.6 | $316.6 | $30.5 | $30.1 | $407.9 | $419.9 |
| Wisconsin | | | | | | |
| Year of Break-even | 2022 | 2019 | 2019 | 2019 | 2020 | 2019 |
| Number Treated | 2,750 | 2,749 | 1,075 | 1,075 | 3,115 | 3,114 |
| Total Benefits | $205.1 | $213.3 | $17.2 | $17.3 | $271.4 | $280.0 |

## State Spending Analysis

In addition to the Markov model described above, we estimated annual federal and state spending based on the total treatment costs and medical expenditures produced by the model. We applied the year- and state-specific Federal Medical Assistance Percentage (FMAP) for Medicaid to the total treatment costs and medical expenditures to determine the federal contribution that year and attributed the remainder of the costs to the state’s spending. The proportion of federal contribution changes year-to-year and is specified for the federal fiscal year (FY). FMAPs are only available through FY 2018. Therefore, we adjust the FY FMAP to the calendar year (CY) for CY 2015-2018 in the model and apply the CY 2018 FMAP to the remainder of the model years. The FY and CY FMAPs by state can be found in eTable 15.

| Table S15. Fiscal year and calendar FMAPs by state[54] | | | | | | | | | |
| --- | --- | --- | --- | --- | --- | --- | --- | --- | --- |
|  | **FY 2014 FMAP** | **FY 2015 FMAP** | **CY 2015 FMAP** | **FY 2016 FMAP** | **CY 2016 FMAP** | **FY 2017 FMAP** | **CY 2017 FMAP** | **FY 2018 FMAP** | **CY 2018 and beyond FMAP** |
| North Carolina | 65.78% | 65.88% | 65.86% | 66.24% | 66.15% | 66.88% | 66.72% | 67.61% | 67.43% |
| Oregon | 63.14% | 64.06% | 63.83% | 64.38% | 64.30% | 64.47% | 64.45% | 63.62% | 63.83% |
| Wisconsin | 59.06% | 58.27% | 58.47% | 58.23% | 58.24% | 58.51% | 58.44% | 58.77% | 58.71% |

# References

1. North Carolina Division of Public Health, *Hepatitis C in North Carolina: Two Epidemics with One Public Health Response*. 2016.

2. Oregon Health Authority, *Viral Hepatitis in Oregon*. 2015.

3. Wisconsin Department of Health Services, *Epidemiologic Profile of Hepatitis C Virus (HCV) in Wisconsin 2014*. 2014.

4. Klevens, R.M., et al., *Estimating acute viral hepatitis infections from nationally reported cases.* American journal of public health, 2014. **104**(3): p. 482-487.

5. Rosenberg, E.S., et al., *Estimation of State-Level Prevalence of Hepatitis C Virus Infection, US States and District of Columbia, 2010.* Clinical Infectious Diseases, 2017. **64**(11): p. 1573-1581.

6. Denniston, M.M., et al., *Chronic hepatitis C virus infection in the United States, National Health and Nutrition Examination Survey 2003 to 2010.* Ann Intern Med, 2014. **160**(5): p. 293-300.

7. *National Hepatitis C Database: for infection acquired through blood and blood products*. 2012, Health Protection Surveillance Centre.

8. Neal, K.R., *Excess mortality rates in a cohort of patients infected with the hepatitis C virus: a prospective study.* Gut, 2007. **56**(8): p. 1098-1104.

9. U.S. Census Bureau. *Annual estimates of the resident population by single year and sex for the Unites States: April 1, 2010 to July 1, 2014*. 2014; Available from: h<ttp://factfinder2.census.gov.> .

10. Lansky, A., et al., *Estimating the number of persons who inject drugs in the United States by meta-analysis to calculate national rates of HIV and hepatitis C virus infections.* PLoS One, 2014. **9**(5): p. e97596.

11. Williams, I.T., et al., *Incidence and transmission patterns of acute hepatitis C in the United States, 1982-2006.* Arch Intern Med, 2011. **171**(3): p. 242-8.

12. Manos, M.M., et al., *Distribution of hepatitis C virus genotypes in a diverse US integrated health care population.* J Med Virol, 2012. **84**(11): p. 1744-1750.

13. Hagan, L.M., M.S. Sulkowski, and R.F. Schinazi, *Cost analysis of sofosbuvir/ribavirin versus sofosbuvir/simeprevir for genotype 1 hepatitis C virus in interferon-ineligible/intolerant individuals.* Hepatology, 2014. **60**(1): p. 37-45.

14. Centers for Disease Control. *Viral hepatitis statistics & surveillance*. 2014 [cited 2014 October 10]; Available from: h<ttp://www.cdc.gov/hepatitis/Statistics/2012Surveillance/index.htm.>

15. Van Nuys, K., et al., *Broad hepatitis C treatment scenarios return substantial health gains, but capacity is a concern.* Health Affairs, 2015. **34**(10): p. 1666-1674.

16. National Institute for Health and Care Excellence. *Final appraisal determination: Ledipasvir-sofosbuvir for treating chronic hepatitis C*. 2015; Available from: h<ttps://www.nice.org.uk/guidance/TA363/documents/final-appraisal-determination-document.>

17. Volk, M.L., et al., *Public health impact of antiviral therapy for hepatitis C in the United States.* Hepatology, 2009. **50**(6): p. 1750-1755.

18. Grabowski, H.G. and J.M. Vernon, *Brand loyalty, entry, and price competition in pharmaceuticals after the 1984 Drug Act.* J Law Econ, 1992. **35**(2): p. 331-350.

19. Arias, E., *United States Life Tables, 2009*, in *National Vital Statistics Reports*. 2014.

20. Mathers, B.M., et al., *Global epidemiology of injecting drug use and HIV among people who inject drugs: a systematic review.* Lancet, 2008. **372**(9651): p. 1733-45.

21. Thein, H.H., et al., *Natural history of hepatitis C virus infection in HIV-infected individuals and the impact of HIV in the era of highly active antiretroviral therapy: a meta-analysis.* AIDS, 2008. **22**(15): p. 1979-91.

22. Hall, H.I., et al., *Prevalence of diagnosed and undiagnosed HIV infection—United States, 2008–2012.* MMWR Morb Mortal Wkly Rep, 2015. **64**(24): p. 657-662.

23. El-Kamary, S.S., R. Jhaveri, and M.D. Shardell, *All-cause, liver-related, and non–liver-related mortality among HCV-infected individuals in the general US population.* Clinical Infectious Diseases, 2011. **53**(2): p. 150-157.

24. McCombs, J., et al., *The risk of long-term morbidity and mortality in patients with chronic hepatitis C: results from an analysis of data from a Department of Veterans Affairs Clinical Registry.* JAMA Intern Med, 2014. **174**(2): p. 204-12.

25. Younossi, Z.M., et al., *Impact of interferon free regimens on clinical and cost outcomes for chronic hepatitis C genotype 1 patients.* J Hepatol, 2014. **60**(3): p. 530-7.

26. Best, J.H., D.L. Veenstra, and J. Geppert, *Trends in expenditures for Medicare liver transplant recipients.* Liver Transpl, 2001. **7**(10): p. 858-62.

27. Micallef, J.M., J.M. Kaldor, and G.J. Dore, *Spontaneous viral clearance following acute hepatitis C infection: a systematic review of longitudinal studies.* J Viral Hepat, 2006. **13**(1): p. 34-41.

28. Thein, H.H., et al., *Estimation of stage-specific fibrosis progression rates in chronic hepatitis C virus infection: a meta-analysis and meta-regression.* Hepatology, 2008. **48**(2): p. 418-31.

29. Dienstag, J.L., et al., *A prospective study of the rate of progression in compensated, histologically advanced chronic hepatitis C.* Hepatology, 2011. **54**(2): p. 396-405.

30. Kanwal, F., et al., *HCV genotype 3 is associated with an increased risk of cirrhosis and hepatocellular cancer in a national sample of U.S. Veterans with HCV.* Hepatology, 2014. **60**(1): p. 98-105.

31. Rein, D.B., et al., *The cost-effectiveness of birth-cohort screening for hepatitis C antibody in U.S. primary care settings.* Ann Intern Med, 2012. **156**(4): p. 263-70.

32. McEwan, P., et al., *The impact of timing and prioritization on the cost-effectiveness of birth cohort testing and treatment for hepatitis C virus in the United States.* Hepatology, 2013. **58**(1): p. 54-64.

33. Kabiri, M., et al., *The changing burden of hepatitis C virus infection in the United States: model-based predictions.* Ann Intern Med, 2014. **161**(3): p. 170-80.

34. Chong, C.A., et al., *Health-state utilities and quality of life in hepatitis C patients.* The American journal of gastroenterology, 2003. **98**(3): p. 630-638.

35. McLernon, D.J., J. Dillon, and P.T. Donnan, *Health-state utilities in liver disease: a systematic review.* Med Decis Making, 2008. **28**(4): p. 582-92.

36. Thein, H.H., et al., *Estimation of utilities for chronic hepatitis C from SF-36 scores.* Am J Gastroenterol, 2005. **100**(3): p. 643-51.

37. Coon, J.T., et al., *Surveillance of cirrhosis for hepatocellular carcinoma: systematic review and economic analysis.* 2007.

38. Saab, S., et al., *Cost-effectiveness analysis of sofosbuvir plus peginterferon/ribavirin in the treatment of chronic hepatitis C virus genotype 1 infection.* Aliment Pharmacol Ther, 2014. **40**(6): p. 657-75.

39. Davis, K.L., et al., *Direct economic burden of chronic hepatitis C virus in a United States managed care population.* J Clin Gastroenterol, 2011. **45**(2): p. e17-24.

40. Gordon, S.C., et al., *Impact of disease severity on healthcare costs in patients with chronic hepatitis C (CHC) virus infection.* Hepatology, 2012. **56**(5): p. 1651-60.

41. Gordon, S.C., et al., *Hepatitis C virus therapy is associated with lower health care costs not only in noncirrhotic patients but also in patients with end-stage liver disease.* Aliment Pharmacol Ther, 2013. **38**(7): p. 784-93.

42. Goodman, Z.D., *Grading and staging systems for inflammation and fibrosis in chronic liver diseases.* J Hepatol, 2007. **47**(4): p. 598-607.

43. Smith, D.J., et al., *Spontaneous viral clearance of hepatitis C virus (HCV) infection among people who inject drugs (PWID) and HIV-positive men who have sex with men (HIV+ MSM): a systematic review and meta-analysis.* BMC Infectious Diseases, 2016. **16**(1): p. 471.

44. Centers for Disease Control and Prevention. *HIV surveillance report, 2011, vol 23*. 2013 [cited 2014 July 25]; Available from: h<ttp://www.cdc.gov/hiv/topics/surveillance/resources/reports/.>

45. Karch, D.L., et al., *Comparative Mortality among People Diagnosed with HIV Infection or AIDS in the US, 2001–2010.* Public Health Reports, 2015. **130**(3): p. 253-260.

46. Kuehne, F.C., et al., *Treatment for hepatitis C virus in human immunodeficiency virus-infected patients: clinical benefits and cost-effectiveness.* Arch Intern Med, 2002. **162**(22): p. 2545-56.

47. Gold, M.R., et al., *Cost-effectiveness in health and medicine*. 1996: Oxford university press.

48. Hornberger, J., et al., *Cost-effectiveness of peginterferon alfa-2a (40kDa) plus ribavirin in patients with HIV and hepatitis C virus co-infection.* J Clin Virol, 2006. **36**(4): p. 283-91.

49. Kim, W. and J.J. Poterucha, *Cost‐effectiveness of interferon alfa 2b and ribavirin in the treatment of chronic hepatitis C.* Hepatology, 2000. **31**(3): p. 807-808.

50. Kim, W.R., et al., *Cost-effectiveness of 6 and 12 months of interferon-α therapy for chronic hepatitis C.* Annals of internal medicine, 1997. **127**(10): p. 866-874.

51. Zeuzem, S., et al., *Glecaprevir–pibrentasvir for 8 or 12 weeks in HCV genotype 1 or 3 infection.* New England Journal of Medicine, 2018. **378**(4): p. 354-369.

52. Vazquez, M., et al., *Efficacy and Safety of Glecaprevir/Pibrentasvir in Patients Co-infected With Hepatitis C Virus and Human Immunodeficiency Virus-1: the EXPEDITION-2 Study.* International Journal of Infectious Diseases, 2018. **73**: p. 372.

53. Asselah, T., et al., *Efficacy of glecaprevir/pibrentasvir for 8 or 12 weeks in patients with hepatitis C virus genotype 2, 4, 5, or 6 infection without cirrhosis.* Clinical Gastroenterology and Hepatology, 2018. **16**(3): p. 417-426.

54. The Henry J. Kaiser Family Foundation. *Federal Medical Assistance Percentage (FMAP) for Medicaid and Multiplier*. 2018 [cited 2018 April 4]; Available from: <https://www.kff.org/medicaid/state-indicator/federal-matching-rate-and-multiplier/?activeTab=graph&currentTimeframe=0&startTimeframe=3&selectedRows=%7B%22states%22:%7B%22connecticut%22:%7B%7D,%22oregon%22:%7B%7D,%22wisconsin%22:%7B%7D,%22north-carolina%22:%7B%7D%7D%7D&sortModel=%7B%22colId%22:%22Location%22,%22sort%22:%22asc%22%7D>.
